# Health equity in the New Zealand health care system: a national survey

**DOI:** 10.1186/1475-9276-10-45

**Published:** 2011-10-20

**Authors:** Nicolette F Sheridan, Timothy W Kenealy, Martin J Connolly, Faith Mahony, P Alan Barber, Mary Anne Boyd, Peter Carswell, Janet Clinton, Gerard Devlin, Robert Doughty, Lorna Dyall, Ngaire Kerse, John Kolbe, Ross Lawrenson, Allan Moffitt

**Affiliations:** 1 School of Nursing, Faculty of Medical and Health Sciences, University of Auckland, Auckland, New Zealand; 2 School of Medicine, Faculty of Medical and Health Sciences, University of Auckland, Auckland, New Zealand; 3 Freemasons' Department of Geriatric Medicine, School of Medicine, Faculty of Medical and Health Sciences, University of Auckland, Auckland, New Zealand; 4 School of Population Health, Faculty of Medical and Health Sciences, University of Auckland, Auckland, New Zealand; 5 Waitemata District Health Board, North Shore, Auckland, New Zealand; 6 Auckland District Health Board, Central Auckland, New Zealand; 7 Waikato District Health Board, Hamilton, New Zealand; 8 Counties Manukau District Health Board, South Auckland, New Zealand

**Keywords:** health equity, Māori, cultural competency, health care system, chronic conditions, cardiovascular disease, chronic obstructive pulmonary disease, congestive heart failure, stroke, diabetes

## Abstract

**Introduction:**

In all countries people experience different social circumstances that result in avoidable differences in health. In New Zealand, Māori, Pacific peoples, and those with lower socioeconomic status experience higher levels of chronic illness, which is the leading cause of mortality, morbidity and inequitable health outcomes. Whilst the health system can enable a fairer distribution of good health, limited national data is available to measure health equity. Therefore, we sought to find out whether health services in New Zealand were equitable by measuring the level of development of components of chronic care management systems across district health boards. Variation in provision by geography, condition or ethnicity can be interpreted as inequitable.

**Methods:**

A national survey of district health boards (DHBs) was undertaken on macro approaches to chronic condition management with detail on cardiovascular disease, chronic obstructive pulmonary disease, congestive heart failure, stroke and diabetes. Additional data from expert informant interviews on program reach and the cultural needs of Māori and Pacific peoples was sought. Survey data were analyzed on dimensions of health equity relevant to strategic planning and program delivery. Results are presented as descriptive statistics and free text. Interviews were transcribed and NVivo 8 software supported a general inductive approach to identify common themes.

**Results:**

Survey responses were received from the majority of DHBs (15/21), some PHOs (21/84) and 31 expert informants. Measuring, monitoring and targeting equity is not systematically undertaken. The Health Equity Assessment Tool is used in strategic planning but not in decisions about implementing or monitoring disease programs. Variable implementation of evidence-based practices in disease management and multiple funding streams made program implementation difficult. Equity for Māori is embedded in policy, this is not so for other ethnic groups or by geography. Populations that conventional practitioners find hard to reach, despite recognized needs, are often underserved. Nurses and community health workers carried a disproportionate burden of care. Cultural and diversity training is not a condition of employment.

**Conclusions:**

There is a struggle to put equity principles into practice, indicating will without enactment. Equity is not addressed systematically below strategic levels and equity does not shape funding decisions, program development, implementation and monitoring. Equity is not incentivized although examples of exceptional practice, driven by individuals, are evident across New Zealand.

## Background

### Health equity

Inequalities preventable by reasonable means are unfair, and in health are indicators of distributional differences in the health status of the population. In all countries, including New Zealand, people experience different social circumstances that result in avoidable differences in health, well-being and length of life. The health system can assist in creating a fairer society and ensuring a fairer distribution of good health. However, this requires a commitment to health equity and evidence-informed action by people at all levels within the health system [1], including those responsible for policy, resource allocation, service provision and measurement. The health care system is recognized as a determinant of health, "influenced by, and influencing, the effect of other social determinants" [2] with an important role to play in promoting health equity. Pursuing health equity means "striving for equal opportunities for all social groups to be as healthy as possible, with selective focus on improving conditions for those groups who have had fewer opportunities" [3].

The New Zealand health system has undergone more than three decades of major restructuring. Since 2000 a greater emphasis has been placed on primary health care [4-6], a strategic health system response to building health equity [7]. A population health approach [8] and social policies directed at closing the health gap have also been features of system restructuring. Chronic illness is the leading cause of morbidity, mortality, and inequitable health outcomes in New Zealand [9,10] and despite aspirations and attempts, the health gap is widening between Māori and non-Māori [11-14]. Māori, Pacific peoples and those with lower socioeconomic status experience much higher levels of chronic disease, earlier in life [15] resulting in higher morbidity and lower life expectancy [16]. The estimated total New Zealand population is 4.4 million people; the European ethnic group comprises the majority of the population (68%), with Māori being the largest minority (15%), followed by Asian (9%) and Pacific (7%) [17].

Currently there is limited data available to measure and compare progress on health equity outcomes across New Zealand District Health Boards (DHBs). The overarching question for this study is whether health services in New Zealand are equitable. We sought to answer this question by analyzing data from a national survey. This survey aimed to identify the extent of evidence-based practices in the chronic condition management of stroke, cardiovascular disease (CVD), chronic obstructive pulmonary disease (COPD), congestive heart failure (CHF) and to a lesser extent diabetes, previously reported [18]. Data were collected on the level of development of components of chronic care management systems, which according to current best medical evidence, must be present to provide best care. Shortfall in provision of these components to any patient is undesirable, and variation in provision by geography, condition or ethnicity can be interpreted as inequitable. This paper reports the findings on equity dimensions: reducing health inequalities; self management support for patients and families; community linkages; aspects of disease management programs that have implications for equity, and strategic approaches to health equity.

The authors argue that New Zealand's approach to health equity is inextricably linked to "who we are as a people" building on a history and culture of promoting human rights and indigenous rights. Therefore, we have included for the benefit of an international audience, a brief historical profile of New Zealand and its relationship to actions on equity, and a description of the New Zealand health care system.

### A history of fairness

Honoring the Treaty of Waitangi signed in 1840 between a Crown representative and over 500 indigenous Māori chiefs sets New Zealand apart - it is the only attempt ever in the world by colonizers and indigenous people to regard a historic document as a living social contract, strengthening a mutual relationship as a basis for nationhood [19]. Fifty years on in 1893, New Zealand became the first self-governing country in the world to grant all women the right to vote in parliamentary elections [20]. Today, almost a third of the elected Members of Parliament are female and in recent years, women have held the country's key constitutional positions: prime minister, governor-general, speaker of the House of Representatives and chief justice [21].

Early attempts to address health and social equity are evident in legislation that revealed attitudes towards welfare. The introduction of a pension for the elderly in 1898, followed by pensions to widows (1911), miners (1915) and the blind (1924) gained New Zealand an international reputation for progressive social policy [22]. In 1900 New Zealand was among the first counties to provide compensation for work injuries with a 'no fault' workers compensation system and was one of the first countries to recognize the social and financial consequences of accidents involving uninsured motorists [23]. The 1930s introduced a movement that led to free education for all people regardless of their ability to pay, academic ability or place of domicile [24]. The 1938 Social Security Act extended the family benefit to all mothers irrespective of the family's income and introduced free health care along with a comprehensive array of welfare benefits [25].

When compared internationally New Zealand ranks highly on human development [26], quality of life [27], life expectancy, literacy, public education, prosperity, the protection of civil liberties and political rights [28] peace [29], economic freedom [30], ease of doing business [31], lack of corruption [32], and press freedom [33].

### Structure of the New Zealand health care system

**Figure 1:**
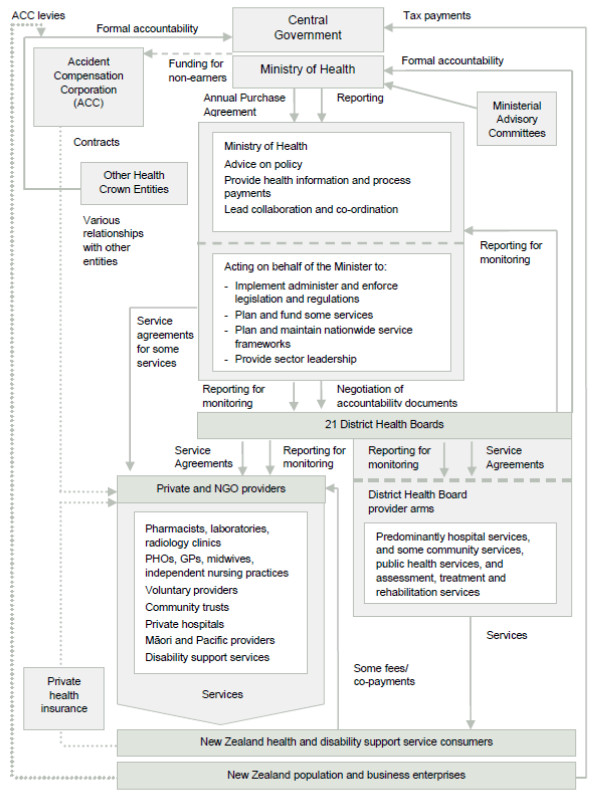
**Structure of the New Zealand health and disability system**. Source: The New Zealand Health and Disability System: Organizations and Responsibilities - Briefing to the Minister of Health, November 2008.

New Zealand's health care system is a mix of public and private ownership across a wide range of health services (see Figure 1) which have experienced radical restructuring over the last three decades. The 1980s saw regional Area Health Board entities with bulk funding of hospitals and other services excluding primary health care. The 1990s saw Crown Health Enterprises, where hospitals were set up as state-owned firms and Regional Health Authorities were purchasers of services, including primary health care. Since 2000, restructuring has occurred within the government elements of the health system. DHBs integrate hospitals into funding bodies. DHBs plan, manage, provide and fund services for the populations of their districts. This includes funding for primary care, public health services, aged care services and services provided by other non-governmental health providers, including Māori and Pacific providers.

DHBs fund the provision of primary health care through Primary Health Organizations (PHOs). Large numbers of PHOs were established between 2002 and 2005; 84 existed by mid-2009. PHOs were tasked to work with local communities and enrolled populations, reduce health inequalities and improve access and provision of services. Changes were made to the method of allocating the public share of primary health care finance from fee-for-service subsidies at the practitioner level to (largely) capitation funding of PHOs [34].

## Methods

The research was conducted under a grant that specified a focus on COPD, CHF, CVD, stroke, with an additional limited data collection on diabetes. Ethics approval was given by the Multi-Region Ethics Committee (MEC/07/21 EXP). A multidisciplinary research team oversaw the project. Members included a senior clinician from each disease area, and people with skills in epidemiology, nursing, primary health care, Māori health, health systems, health management, and evaluation methodology. A literature review was followed by an assessment of long term condition management across the country. The data reported in this paper was collected using two methods, survey questionnaires to DHBs and PHOs and expert informant interviews on chronic condition management. No patients were interviewed.

### Survey questionnaires

The literature review identified ten dimensions of long term condition management. We selected three dimensions: reducing health inequalities; self management support for patients and families; and community linkages as relevant to health equity. Other dimensions not reported are: conceptual understanding, effective leadership, implementation of guidelines, collaboration, delivery system design, decision support, and knowledge transfer.

The survey tool comprised seven separate questionnaires:

1. Chronic condition management

2. COPD

3. CHF

4. CVD

5. Stroke

6. Primary health care

7. Health inequalities

Questionnaire one, chronic condition management comprised the dimensions cited above. The three dimensions reported in this paper are represented by a series of statements where respondents rated their organization on a scale ranging from 0-11. General descriptors represent grouped numbers: 0-2 "little support", 3-5 "basic support", 6-8 "good support" and 9-11 "full support". With each of these broad groupings further text, specific to each item, described what was meant by little, basic, good or full support. For example, the first item under "inequalities in health care" defined little support as "does not exist or there is little interest"; basic support as "is reflected in vision statements and high levels of system plans, but resources are not dedicated to programs aimed at reducing inequalities"; good support as "is reflected in senior leadership and resources dedicated to programs aimed at reducing inequalities"; and full support as "is part of the system's long term planning, resources are dedicated to programs at all levels of the organization, specific people are held accountable for the implementation and monitoring of programs and outcomes".

Questionnaires two to five use the same format and each has a single disease focus on COPD, CHF, CVD and stroke respectively. Questions ask about the characteristics of populations accessing services, tests, smoking cessation, staffing levels, culturally specific programs, self management, education, inpatient services including rehabilitation, discharge services including outpatient, case management, tele-monitoring, palliative care, and resourcing.

Questionnaire six, primary health care comprised six sections, one general and five focusing on COPD, CHF, CVD, stroke and diabetes respectively. Respondents rated their organization on a scale of 0 to 11 after reading a series of statements about program availability, group education for patients and families, outreach, shared records, culturally specific programs, nurse-led clinics, case management, and community health worker roles. The scores were again in four broad groupings: 0-2, 3-5, 6-8 and 9-11 against descriptive anchors that were specific to each statement item. For example, the item "programs to care for patients with CVD", included text that described responses 0-2 as "no formal program"; 3-5 as "program within practice of PHO"; 6-8 as "program developed by primary and secondary care, regular information transfer, feedback of specialist advice to primary care"; and 9-11 as "program formalized along the Chronic Care Model or equivalent (includes decision support, patient self-management, system process redesign). This item has the same format for COPD, CHF, stroke and diabetes.

Questionnaire seven, health inequalities focused on organizational macro-level strategy. Questions ask about frameworks and policies used to plan and implement action on health inequalities, resources committed to ensuring outcomes for groups at greatest disadvantage, existing inequalities, identifying the most disadvantaged, identifying and quantifying issues of access into programs for people with chronic conditions, assessing barriers to access and strategies to reduce/eliminate these barriers, and cultural safety training for staff.

The survey tool (comprising all seven questionnaires) was reviewed by an expert panel and piloted in two DHBs in 2007. Minor refinements were made and included clarification of the ICD 10 codes (International Statistical Classifications of Diseases, version 10 was updated in 2009) for use in COPD, CHF, CVD and stroke when extracting data from patient information systems and the use of discharge rather than admission data to code events. In October 2007 the 21 DHBs were contacted before the survey was mailed to a clinical leader, designated by the Chief Executive or equivalent, to coordinate a response. No one person in the DHB was thought to have the breadth of knowledge to answer all survey questionnaires. Consequently, senior clinicians, Māori general managers, Pacific and Asian general managers, and senior funders and planners contributed to this response. Non-respondents were followed up by phone and letter.

The focus of enquiry centered on DHB perceptions, rather than those of PHOs, consumers or other parties. Despite this main focus, all 84 PHOs were additionally mailed the chronic care management primary health care questionnaire and the health equity questionnaire. Due to constraints on project resources PHO non-respondents were not followed up.

### Expert informant interviews

Ten expert informants were selected by the research team on the basis of national prominence in long term condition management, ethnicity, occupation, employer, and geography. Subsequent informants were identified through recommendations made by initial informants. Consent was obtained from each person before the interview was undertaken. Face-to-face and telephone interviews lasting up to 60 minutes were conducted in English. The interview questionnaire sought perspectives on chronic care as well as the challenges and enablers to management. Of specific interest were programs targeting people labeled hard to reach and cultural responsiveness in meeting the needs of Māori and Pacific peoples.

### Data analysis

Descriptive statistics prepared in Microsoft Excel are presented from the numerical response sections of the questionnaires. Open text responses were summarized. The expert informant interviews were transcribed and NVivo 8 software was used to support a general inductive approach to identify common themes [35]. Data from the surveys and the expert informant interviews were combined so that each topic was informed from both data sources. In particular, qualitative data provided context for the quantitative summary data.

## Results

Survey data were received from 15 of 21 DHBs and 21 of 84 PHOs. A clinician leader, designated by the Chief Executive or equivalent, coordinated the organizations single response, which required different people within the organization contributing specific knowledge. DHBs and PHOs were representative of geographic regions (urban and rural; north and south islands), population size and ethnic composition.

Interview data from 31 expert informants with knowledge of chronic condition management in primary health care was obtained. Of these, 19 were female and six were Māori. All but two had backgrounds in nursing, medicine or pharmacy. In addition, most were also members of PHO or Health Boards. Employers included the Ministry of Health, PHARMAC (Government Pharmaceutical Management Agency), DHBs, PHOs, Māori Provider Organizations, Universities, or were self employed in general practices. Key themes from interview data were: targeting equity groups; developing (Māori) nursing and community health worker services; the need for data to measure equity in service provision; lack of cultural safety and diversity training; and barriers and enablers to improving access to health care services.

### Strategic focus on equity

Thirteen of 14 DHBs reported use of the Health Equity Assessment Tool (HEAT) [36,37] at the population level. Four DHBs had identified inequalities in strategic or business plans. Other health plans were developed for defined populations: Māori (13); Pacific (7) and Refugee and Migrant (1); no DHB had developed an Asian Health Plan. Two DHBs nominated Older Persons, Primary Health Care, Palliative Care and Chronic Conditions plans as related to reducing inequalities. Several expert informants stated the importance of plans that could guide macro-level decisions affecting the whole system. One person said, *"All programs should be developed on an equity basis... use of the HEAT tool from design and development would identify what needs to be done differently for groups" *(GP Clinical Liaison, Pakeha [NZ European]).

Twelve DHBs had committed resources to ensuring that the most disadvantaged groups benefited earliest and most significantly. They pointed to programs that were often not specific to one condition, chronic conditions or specific disadvantaged populations. Examples included: housing projects; health promotion and health care clinics in low decile schools; dental care for those on low incomes; oral healthcare for people with mental health issues; funding for asthma, diabetes and mental health in Pacific primary care; free general practice consultations; free interpreter services; outreach diabetes and respiratory services, and mobile Māori nursing services. An expert informant said, *"We have mobile nursing services and we can actually go to people in their homes. We can also relate to them as we are Māori from this area, so we have affiliations... they are our whanau (family)" *(PHO Nurse, Māori).

### Chronic care management programs

Fifteen DHBs and 21 PHOs responded to questions about chronic care management reported in Tables 1 and 2 respectively. Three dimensions: community linkages, inequalities in health care, and self-management support were each queried by four or five items. Median scores from DHBs equated to 3-5 "basic support" or 6-8 "good support" and from PHOs equated to 6-8 "good support" or 9-11"full support". However, the wide variation between DHBs and between PHOs suggested inequity by region.

**Table 1:** Summary of DHB responses to questions about chronic care management

	Median (Range)
**Inequalities in Health Care**	

Strategic focus to reduce inequalities	8 (4-11)

Commitment to Māori and developing cultural safety	8 (3-10)

Commitment to cultural safety when working with people diverse in ethnicity, religion, sexual preference, and with different physical and mental abilities	6 (2-9)

Level of equitable access to health care	6 (3-9)

**Community Linkages**	

Linking patients to outside resources	7 (2-9)

Partnership with community organizations	6 (4-11)

Traditional healers and complementary alternative therapists	4 (1-7)

Biculturalism as a continuum with a graduation of goals and a number of possible structural arrangements	8 (3-10)

Partnerships with consumers	7 (2-9)

**Self Management Support**	

Assessment and documentation of self management needs and activities	4 (0-10)

Self management support	5 (0-10)

Addressing concerns of patients and families/whanau	5 (2-10)

Effective behavior change interventions and peer support	6 (2-10)

Patient engagement with the chronic care management program	4.5 (2.5-10)

N = 15 DHBs. Responses to each item were scored on a scale from 0-11. Scores 0-2 indicate "little support", 3-5 "basic support", 6-8 "good support" and 9-11 "full support".

**Table 2:** Summary of PHO responses to questions about chronic care management

	Median (Range)
**Inequalities in Health Care**	

Strategic focus to reduce inequalities	9 (4-11)

Commitment to Māori and developing cultural safety	9 (3-11)

Commitment to cultural safety when working with people diverse in ethnicity, religion, sexual preference, and with different physical and mental abilities	9 (3-11)

Level of equitable access to health care	9 (3-11)

**Community Linkages**	

Linking patients to outside resources	6.5 (3-11)

Partnership with community organizations	8 (3-11)

Traditional healers and complementary alternative therapists	6 (2-10)

Biculturalism as a continuum with a graduation of goals and a number of possible structural arrangements	8 (3-11)

Partnerships with consumers	7 (3-11)

**Self Management Support**	

Assessment and documentation of self management needs and activities	7 (0-11)

Self management support	5 (0-11)

Addressing concerns of patients and families/whanau	6 (3-11)

Effective behavior change interventions and peer support	8 (3-10)

Patient engagement with the chronic care management program	6 (0-11)

N = 21 PHOs. Responses to each item were scored on a scale from 0 to 11. Scores 0-2 indicate "little support", 3-5 "basic support", 6-8 "good support" and 9-11 "full support".

Chronic condition management programs included depression, obesity and asthma as well as COPD, CHF, CVD, stroke and diabetes. Seven programs had a single disease focus and ten were multi-disease. We asked about each of our index conditions and about the processes used to support people enrolled with the organization by ethnicity and quintile. Many of the programs appeared to be capable of addressing long term conditions and were need or symptom focused. Nurses with extended roles were prominent in programs; ethnic group focused almost solely on Māori, and most provided care across all deprivation quintiles.

### COPD, CHF, CVD, stroke and diabetes

Twelve DHBs responded to questions about the five conditions reported in Table 3. This data addresses more than one question. Variation in the median scores between chronic conditions suggests inequity by condition. Diabetes services were consistently the most developed (except for community health workers, median scores were 4.5-7 out of a possible 11) and stroke services were consistently the most poorly developed (medians 1-4). Across all four conditions the least developed service components were community health worker services (medians 0-2, the highest being diabetes), ethnic specific services (medians 0-3.5 expect for median 6 for diabetes) and outreach services (medians 1.5-4.5, the highest being for diabetes). The wide range of scores between DHBs, by condition and by service component, implies regional inequity. In addition, text responses to questions on the four conditions (excluding diabetes) are reported.

**Table 3:** Summary of DHBs responses to questions about primary care by condition

	CHF Median (Range)	CVD Median (Range)	COPD Median (Range)	Stroke Median (Range)	Diabetes Median (Range)
Programs to care for patient with (condition)	5 (0-11)	5 (2-10)	3.5 (0-11)	2 (0-11)	7 (4-11)

Support for group education and consultations for patients with (condition) and their family/whanau	2.5 (0-5)	3 (0-7)	3 (0-7)	1.5 (0-4)	4 (0-8)

Outreach programs for people with (condition)	2 (0-9)	1.5 (0-9)	2 (0-6)	1 (0-10)	4.5 (0-8)

Shared records for (condition)	3 (0-8)	4.5 (0-9)	3 (0-7)	3 (0-7)	5 (1-7)

Ethnic/culture specific programs for (condition)	0 (0-5)	3.5 (0-8)	1 (0-8)	1 (0-4)	6 (0-8)

Nurse led clinics for (condition)	5 (0-9)	5 (0-7)	3.25 (0-8)	4 (0-10)	7 (4-10)

Case/care management for (condition)	4.5 (0-7)	4.5 (0-7)	4 (0-7)	4 (0-10)	5 (4-10)

Community Health Workers (unregulated workers) for (condition)	0 (0-4)	1 (0-6)	1 (0-7)	1 (0-10)	2 (0-8)

Absolute risk assessment for CVD		8 (2-10)			

Local or regional disease register for Diabetes					6.25 (2-10)

N = 12 DHBs. Responses to each item were scored on a scale of 0 (no development) to 11 (full development). Results are Median (Range).

COPD: Fifteen DHBs answered this section. Twelve offered a pulmonary rehabilitation program, of which five included a community base. Four ran a home based exercise program; five offered other community services, and four surveyed patients' experiences of the rehabilitation service. Two offered a hospital at home service for acute exacerbations of COPD. Thirteen had palliative care support. Nine offered self management programs, of which seven included written action plans. Three DHBs offered nurse case management as an outreach service from secondary care and included home visiting. Two DHBs offered ethnic-specific COPD programs, with nine having cultural support workers to assist patient access.

CHF: Fourteen DHBs answered this section. Three provided hospital at home services with small numbers. Twelve offered outpatient CHF management; ten were home based services. In nine DHBs this included a self management program with written action plans in seven. Eight offered case management for their most complex patients. Five DHBs integrated services across secondary care, primary care and patients' homes and the most pro-active DHB invoked this service when a patient had two or more hospital admissions in one year. Although 11 provided palliative care for patients with end-stage CHF, this appears well developed across health sectors in only three DHBs with one specifically noting gaps in the service for Māori and Pacific patients. Two DHBs provided ethnic specific CHF programs, and ten had cultural support workers to support clinical care. Nurse led clinics, a formal chronic care management program in primary care, and a health psychologist within the cardiology service were additional services described by one DHB.

CVD: Thirteen DHBs answered this section. Twelve provided a cardiac rehabilitation service, of which six were hospital based, two community based and four were both. Of these, three surveyed patients' experience of the rehabilitation service. Nine DHBs offered a self management program with written action plans in eight. Six DHBs provided case management for the most complex patients and one provided this routinely to all patients. One DHB provided an ethnic specific CVD program and nine had cultural support workers to support clinical care. Cultural support for Māori included DHBs funding new positions for Māori community health workers, nurse clinics, and health promotion in primary care.

Stroke: Fourteen DHBs answered this section. Ten provided a driving assessment service, five being free to at least some patients. Only one DHB provided an ethnic specific program, although 13 had cultural support workers to support clinical care.

Twenty-one PHOs responded to questions about the five conditions reported in Table 4. As with the DHB responses, data shows diabetes services to be the most developed and stroke the least. Diabetes medians were 6-9 out of a possible 11 (except for a median of 3 for community health workers), while stroke medians were 2-4. Also similarly to DHBs, PHO scores were lowest for ethnic specific services, community health workers and outreach programs. The ethnic specific service median was 2 for CHF, COPD and stroke, with 5 for CVD and 6 for diabetes. The outreach services median was 2 for all services except 6 for diabetes. The community health workers median was 2-3 across all services. As with DHBs, the wide range of responses between PHOs implies regional inequity.

**Table 4:** Summary of PHOs responses to questions about primary care by condition

	CHF Median (Range)	CVD Median (Range)	COPD Median (Range)	Stroke Median (Range)	Diabetes Median (Range)
Programs to care for patients with (condition)	3 (1-11)	7 (0-11)	4 (0-11)	2 (0-11)	9 (2-11)

Support for group education and consultations for patients with (condition) and their family/whanau	4 (0-11)	5 (0-11)	4 (0-11)	2 (0-7)	8 (2-11)

Outreach programs for people with (condition)	2 (0-8)	2 (0-11)	2 (0-11)	2 (0-5)	6 (0-11)

Shared records for (condition)	3 (0-11)	6 (0-11)	4 (0-11)	4 (0-11)	7 (2-11)

Ethnic/culture specific programs for (condition)	2 (0-8)	5 (0-11)	2 (0-11)	2 (0-7)	6 (0-11)

Nurse led clinics for (condition)	4 (0-11)	6 (0-11)	5 (0-11)	2 (0-7)	8 (2-11)

Case/care management for (condition)	4 (0-11)	5 (0-11)	4 (0-11)	3 (0-10)	8 (1-11)

Community Health Workers (unregulated workers) for (condition)	2 (0-10)	3 (0-10)	2 (0-11)	2 (0-7)	3 (0-11)

Absolute risk assessment for CVD		7 (1-11)			

Local or regional disease register for Diabetes					9 (2-11)

N = 21 PHOs. Responses to each item were scored on a scale of 0 (no development) to 11 (full development). Results are Median (Range).

### Improving access - nurses, community health workers and new initiatives

A national initiative "Care Plus" targeting better coordinated and lower cost services for people with complex or chronic conditions was acknowledged by all respondents as having made an early impact on both systems and care. Several expert informants commented that Care Plus funding had created an opportunity for practice nurses to expand their roles. There was general agreement that the health system focus of long term condition management had resulted in a widespread development of nursing services. These included outreach, clinical case management, the chronic care family nurse, and Māori nurse disease-state management roles. Similarly, an increased emphasis had been placed upon the community health worker role. Within multidisciplinary teams, including general practice physicians, specialist physicians, psychologists, podiatrists, dieticians, and social workers, nurses and community health workers were seen as having central and complementary functions.

Māori nurses and kaiawhina (Māori community health workers) were integrating kaupapa (first principles) when engaging with Māori clients. A nurse explained, *"Through our kaupapa, Māori are working with Māori to manage their chronic conditions, we walk alongside clients and their whanau" *(Māori Provider Organization, Nurse, Māori). A kaiawhina role was described as assisting patients to access housing and welfare (social) services and (re) engage with general practice. One person said, *"the kaiawhina will determine (problems) through a home visit, it could be simple such as lack of transportation" *(PHO Nurse, Pakeha).

A Māori PHO in an area of high deprivation offered free medical and nursing care with home visiting, charging only a small cost for medications. In contrast, a PHO offering medical outreach in an urban area was unable to extend the service to rural enrollees because of cost. Health education was typically traditional one-on-one conversations, although examples illustrated group education with patients and families occurring in a wider range of settings, such as on marae (Māori meeting place), church halls, mobile nurse clinics and patients' homes. Other initiatives to improve access into primary or community services included green prescription (referral of a patient by a nurse or doctor for exercise or lifestyle activity), family lifestyle programs, smoking cessation programs, men's health checks aimed at Māori, Pacific and men from low socio-economic backgrounds, rural or disease specialists in primary care, interpreting services, employing Māori receptionists; cultural brokers (whanau support workers); vouchers for free GP visits on hospital discharge, online consultations, incentivized disease coding in primary health care to develop disease registers, implementing information systems, establishing audit and feedback systems, alliances with Māori provider organizations, and with Pacific church leaders case finding people known, but not responding to GP recalls.

### Measuring, monitoring and targeting equity

When DHBs were asked whether they had data on the number of people with specific conditions by ethnicity and quintile, eleven indicated they had such data for diabetes, four for COPD and stroke, and two for CHF and CVD. Available data related mostly to utilization of secondary care services, disease-specific outreach services, or was collected by PHOs. Insufficient information was a main concern with one person stating, *"Without information you cannot reliably manage anything. If you want to reduce inequalities first of all you need to make sure you have got the information that tells you where the gaps are. We don't know enough to effectively engage with these people..." *(GP Medical Advisor, Pakeha). Another argued that even when patients had been using services they had limited information, often making it impossible to follow-up. *"There are the people who have stopped engaging with us, they have DNA (did not attend) beside their names, or they have moved from us but didn't actually go anywhere else" *(Māori Provider Organization GP, Pakeha). One PHO manager explained that many who were underserved were not visible to primary care, particularly if they were not enrolled in a PHO, and said *"(we) report on who completes the program... we don't do enough in up front tracking, or determine why people don't come, we confine our successes to those who come" *(PHO Manager, Māori).

Nevertheless, DHB survey respondents identified populations subject to inequalities and disadvantage - Māori, Pacific, low socioeconomic quintile, low income workers (who had difficulty accessing health service during working hours), rural, elderly, disabled, migrants, refugees, those with poor English language skills, and those living in specified localities. Whilst expert informants identified the same populations, often describing them as hard to reach, they described prioritizing health interventions on limited data about their populations. Most thought the number of people underserved was grossly underestimated and that the hardest populations to connect with were the least known about and understood.

There was limited knowledge in the sector about ethnicity and quintile breakdown of populations with either undiagnosed disease or who did not attend secondary services. One DHB made specific mention of ongoing problems with accuracy of ethnicity recording in hospital data. Several expert informants identified cultural responsiveness as a quality indicator of service provision and the importance of ethnicity data to quantify actual numbers of clients and to assess cultural acceptability and appropriateness of services.

### Cultural safety or diversity training

Nine DHBs required employees to undertake cultural safety training in relation to Māori, although numbers indicated that only a minority of staff had undertaken such training at any one time. In contrast, 17 PHOs required employees to undertake cultural safety training in relation to Māori, and reported that the majority of their staff had undertaken such training. Only one DHB required employees to undertake training in the areas of disability, ethnic, cultural/religious, sexuality and gender diversity, whilst 11 PHOs had not required this training. No DHB had data on the number of employees who had undertaken cultural safety or diversity training. PHO estimates of the actual percentage of employees who had undertaken diversity training ranged from unknown or ten percent to all employees.

A Māori manager argued, *"Cultural competence is about awareness... not just about Māori and Pacific Islanders... it's about everyone" *(PHO Manager, Māori). Expert informants collectively stated that chronic care programs were inadequate in meeting the cultural needs of diverse groups, but were mostly concerned with Māori and/or Pacific peoples. Several expert informants commented that many programs were Euro-centric and poorly adapted for Māori and Pacific peoples, risking failure. Concerns surrounded culturally inexperienced clinicians who had little or no training and awareness and the lack of ongoing cultural advice or support that was available within organizations. One person said, *"we may not be arming our champions with the types of skills, resources, training or tools in their kete (basket) to work and motivate people to engage in programs" *(PHO Manager, Māori).

Most expert informants discussed the need for a health workforce that reflected the population ethnic demographic and the need to build this capacity within Māori, Pacific and Asian ethnic groups. The notion of "by Māori for Māori" was widely accepted, but not seen as the only strategic approach. Working in partnership with Māori or other ethnic groups was not disputed, although most informants believed this was not routine practice. Successful engagement was seen as critical, *"You might keep the same overall principles of what makes a good chronic care management program, but in consultation with the community you adopt what will work best" *(Medical Advisor, Pakeha).

### Barriers and enablers to improving access

PHOs were asked about difficulties patients might have accessing programs for long term condition management, and what they were doing to address these issues. Sixteen PHOs said they had or were developing systems to identify patients with access difficulties in order to quantify this issue and provided lists of barriers and processes to improve access. Major barriers reported at the provider level included a lack of dedicated nursing time to provide services needed, limited access to interpreting services and community advisors, improving data collection and analysis and administrative processes around recall systems.

Barriers for patients were repeatedly cited as transport, costs, low health literacy, language and cultural barriers and the low priority given by patients to their chronic conditions. Several expert informants spoke of the need to better understand the experience of living with an illness. A nurse commented *"We need to talk to communities to determine how they are managing chronic conditions in the context of their lives right now... we need to focus on peoples' strengths" *(Pacific Nurse, Samoan). Another said, *"unless you address the patients perceived barriers to self management things are not going to work... unless you establish what might be stopping them from taking an active role in their self management and address that you might as well be talking to a brick wall" *(GP Medical Advisor, Pakeha).

Community governance was considered an enabler to a successful program. One expert informant asserted *"Having significant governance, absolutely not nominal governance... contributory leadership.... to deliver to the bottom, where people have been intimately involved in agreeing" *(GP, Māori) whilst others contended it was necessary to have full control - by Māori for Māori.

Expert informants reported greater cost and more effort were deterrents for many PHOs committing to chronic care management programs. One person stated *"it is much easier to develop chronic care management programs for the people who actually comply with what you say, we get paid the same amount of money" *(GP Medical Advisor, Pakeha). Others thought program funding was difficult to use flexibly, although one person said "*it is a process of adapting and being flexible in how you deliver the program" *(PHO Nurse Manager, Pakeha). This was consistent with examples that pooled funding from different programs targeting clients in the same family, such as Whanau Ora (family health) and chronic condition management, where case management approaches that centered on families were described.

## Discussion

Māori indigenous rights have been at the forefront of political debates since the signing of the Treaty of Waitangi where "partnership" was guaranteed. Biculturalism [38] has challenged New Zealand society to face race equity and the authors argue that this stimulated societal recognition of human rights and a wider diversity culture. The right to health is the right to "the highest attainable standard of health" [39] which means that the health levels of the most privileged groups in a society should be attainable by everyone in that society [40]. In New Zealand health equity is firmly embedded in national health care policies, although despite recognition of other targeted groups, Māori is the population most explicitly prioritized because of Treaty status [4,6]. New Zealand like most countries is struggling to achieve vertical equity, whereby people with greater health needs receive more health care services [41]. Using data from a national survey we have been able to describe the current state of chronic care management in New Zealand, but our data are not adequate to describe progress in the sense of improvement over time, and do not measure outcomes. Indeed, this paper is part of our advocacy for systematic measuring, monitoring over time, and action to support a policy of closing equity outcome gaps.

Diabetes services were consistently the most developed and stroke services were consistently the most poorly developed (with possible implications of inequity by age). Diabetes has been a focus for priority attention in New Zealand for nearly two decades [16]. Variation in medians between components of a service - with ethnic specific services, community health worker services and outreach services being consistently poorly developed, indicates a health system that remains narrowly oriented to a 'medical model' of provision rather than one that comprehensively addresses the wider determinants of health. The variation between DHBs and between PHOs was striking, both by condition and by service components, implying substantial regional inequity.

The large number of DHBs and PHOs in New Zealand reflects the desire for local responsiveness in decision making however, there is also a need for national policy and monitoring to ensure non-negotiable issues such as equity remain in focus. We found detailed planning and implementation of health care policy was devolved to DHBs who often further devolved responsibility to PHOs. Although health equity was recognized in DHB strategic documents, it was not addressed systematically below this level. Thirteen of 14 DHBs used the HEAT tool to guide strategy. In practice this tool was used to focus on health equity for Māori. There was limited evidence in policy or practice of health equity being addressed for Pacific or Asian minorities, for people on low-incomes or by geography. Services as delivered varied from non-existent to exemplary across any equity issue. Slow translation of policy into practice and limited measuring and monitoring of health equity was characteristic. There were no incentives to manage health equity and there was limited use of health equity to shape funding decisions, program development and care delivery.

At the level of geographic or postcode equity, it is striking that provision of basic, evidence-based disease-specific services such as pulmonary rehabilitation and cardiac rehabilitation and stroke units were not consistently provided around New Zealand. In the absence of processes to manage equity, service delivery can only be driven by the particular interests of those leading health organizations, of senior clinicians and of those using health services including organized community networks. Disease specific programs run by local specialists varied in terms of the presence or absence of evidence-based chronic care program components. For example, not every DHB had a pulmonary rehabilitation program for COPD patients that included all the evidence-based components. Quality programs for specific issues in specific localities that depend on local specialists may not be sustainable and the likelihood that such programs will become generalized is also not assured. In addition, lists of programs that were not disease-specific, such as Care Plus, were provided by respondents who described marked variability. Regional variation at best can identify new improved ways of working, such as telemedicine, but only if the health system has systematic evaluation built in, along with systematic processes to disseminate innovations.

Expert informants stated that the system often failed to act in the face of known inequity. Starfield strongly argues "inequity is built into health systems" [41], therefore our challenge must be to build equity into our health system. Given the examples of equitable performance already present in New Zealand it is clear that, as the American-Canadian writer and futurist William Gibson said "The future is already here. It's just not very evenly distributed" [42]. What is currently exemplary must become the norm.

Firstly, it must be recognized that inequity is a complex problem that has been termed "wicked" [43]. Wicked problems: are difficult to clearly define; have many interdependencies and are often multi-causal; are often not stable; usually have no clear solution; are socially complex; hardly ever sit within the responsibility of one organization; involve changing behavior; are often characterized by chronic policy failure; and attempts to address them often lead to unforeseen consequences. Addressing the wicked problem of health inequity requires a whole of government approach and constructing and managing a collaboration of the many interested parties. The required system levers include policy, funding, contracts, monitoring, attitude and culture change [44], education and regulation.

Whilst high level health equity national policies are already in place they will need continuing refinement as progress is made. The next issue, and probably the most immediately achievable, is that policies need to be actively monitored and defined action taken in response to results. This monitoring should be reflected in contracts which control funding. Such monitoring is needed at each level of the system, both self-monitoring and monitoring of multiple layers in the system: Ministry of Health monitoring DHBs; DHBs monitoring PHOs; PHOs monitoring practices. Current monitoring of equity is largely based on secondary use of data collected for clinical or administrative purposes. In contrast, Braveman [3] and Whitehead [45] describe the process required for routine equity monitoring as one that requires the identification of an indicator of health or a modifiable determinant and an indicator of social position, before measuring the first across strata of the second. In the process one also needs to consider the possibility of harm by assessing differential impacts between and within social groups, eschewing "averages" which can mask disadvantage.

Multiple funding streams were repeatedly identified by expert informants as a barrier to innovative health equity solutions. These can be difficult to address, requiring collaboration across silos within or between organizations. Senior people responsible for funding decisions can learn from working with others who are maximizing resources by aligning programs, reducing service duplication and focusing on health equity.

In the longer term, addressing health equity needs to include undergraduate education, attitude and behavior change of those in the health care system, and citizens. Equality of access is inequitable in the face of unequal need [41]. The slow penetration of equity into practice may stand mute testimony to an undercurrent of reluctance on the part of health workers to enact positive discrimination. Our expert informants suggested that some New Zealanders would limit notions of fairness to equality of service access. Such a view disregards clear evidence that opportunities are not equally distributed and in the absence of "leveling up" programs, already advantaged groups consistently get more of the resource [46]. The necessity for attitude and behavior changes of health service personnel is consistent with the "informal power" of frontline workers to undermine services they do not actively support [47] and the inevitable process of policy adaptation-in-application by frontline workers or "street level bureaucrats" [48].

It is clear from our interviews and wider evidence [49] that cultural competency is a requisite component of quality health care, as assessed by patients, and in association with good health outcomes. Cultural competency is mandated by the Health Practitioners Competence Assurance Act 2003 [50] and is further defined by the Medical Council of New Zealand [51] and the Nursing Council of New Zealand [52]. Cultural competency training was a condition of employment in the three Māori PHOs, and was required by the majority of DHBs and PHOs although no accurate data were available on numbers of employees that had undertaken training. Expert informants identified cultural competence as a significant health workforce issue, but it is unclear whether specific training programs improved and sustained cultural competency, a necessary capability for professionalism and quality.

We are encouraged by the ubiquitous mention of community health workers, such as kaiawhina, who at best might be filling the cultural and equity gaps not met by current programs. Even though this role is widely acknowledged as important by DHBs, PHO ratings indicated these roles as the least well developed aspect of their disease specific programs (see Table 4). Nursing services providing outreach or offering mobile disease-state (case) management in Māori communities were rated highly by expert informants as was the training of health workers from the same ethnic groups as those experiencing inequity.

### Limitations

The responses to our survey questionnaires were incomplete despite the study being funded from the national DHB organization, which required responses from members' staff. We acknowledge that many, especially smaller DHBs do not have the analytic staff to either respond themselves or provide the level of information required to the relevant clinical leader who was tasked to respond. Since these data were collected there has been some movement to amalgamate DHBs and similarly for PHOs, in an attempt to pool analytic and policy support and reduce administrative costs across a smaller number of organizations. The PHO response was particularly limited by our lack of resources to make contact prior to questionnaires being posted and to follow initial non-responders. Despite this, responses from PHOs were surprisingly consistent with those from DHBs and expert informants.

The equity issues we report here come from data collected within a wider survey. The questionnaires were completed by several people in a DHB or PHO with a different person responding to questions related to their responsibilities within the organization. Responses should be treated as self-report data by the most relevant people in organizations. In practice responses to open ended text sections of the questionnaires are likely to under-report, for example, the range of programs operating. Nevertheless, clear patterns emerge after collecting data from multiple sources and correlating survey and interview data.

## Conclusions

New Zealand has a history of taking action to achieve equity. Currently in health policy however, we are stating intentions to reduce inequities without systematically applying evidence to the management and monitoring of chronic conditions; this is not enough. National and regional attempts to achieve health equity have largely focused on Māori with relatively less attention to Pacific and Asian minorities, people on low incomes or by geography. There is a need to transfer to other populations' policy mechanisms and implementation methods that have underscored success for Māori. An equity approach must be institutionalized throughout the health sector and beyond to other sectors that impact on health such as education, social welfare, and housing.

Study participants recognized the importance of formal requirements to address inequity such as mandating the use of the HEAT tool in planning; requiring all evaluations to assess the impact on inequity and requiring DHBs to report on their progress as part of the monitoring of their contracts. There is a critical need for sustained health sector leadership in tackling inequalities and challenging the willful ignorance embedded in habitual, inequitable practices. For those who work in the health sector cultural and diversity training provides an opportunity to question assumptions, deepen thinking, and inform safe practice. It is an organizational responsibility to set frameworks that can guide development, implementation and monitoring of cultural competency in the workforce. As well, good policy needs to distinguish between providers that are doing good and providers that are doing harm [53].

Health equity is an international priority [41]. Too many governments know too little because they do not systematically measure equity in their populations. This study has offered a snapshot of equity policy and practice in the New Zealand health system. Internationally, we have some lessons to offer, but we have a long way to go.

## Competing interests

The authors declare that they have no competing interests.

## Authors' contributions

NS and TK conceived the idea, collated and analyzed the data and wrote the paper. FM collected and summarized data and helped draft the manuscript. MC was principal investigator of the larger study within which the current study is nested and helped draft the manuscript. All authors contributed to the study design and helped draft the manuscript. All authors read and approved the final manuscript.

## Authors' information

NS is Associate Dean Equity, Faculty of Medical and Health Sciences, The University of Auckland. MC, AB, GD, RD, JK are specialist hospital-based physicians; TK, NK, RL, AM are primary health care physicians; NS is a nurse; PC is an organizational psychologist; JC is an evaluator; MB is a senior manager, innovation; LD is a public policy expert; FM is a senior researcher. NS, AB, LD, NK are of Māori descent.
